# Effect and Potential Mechanism of Electroacupuncture Add-On Treatment in Patients with Parkinson's Disease

**DOI:** 10.1155/2015/692795

**Published:** 2015-08-13

**Authors:** Fang Wang, Li Sun, Xiao-zhe Zhang, Jun Jia, Zhuo Liu, Xi-yan Huang, Shu-yang Yu, Li-jun Zuo, Chen-jie Cao, Xiao-min Wang, Wei Zhang

**Affiliations:** ^1^Department of Neurology, Beijing Tiantan Hospital, Capital Medical University, Beijing 100050, China; ^2^Department of Acupuncture, Beijing Tiantan Hospital, Capital Medical University, Beijing 100050, China; ^3^Department of Physiology, Capital Medical University, Beijing 100069, China; ^4^Department of Geriatrics, Beijing Tiantan Hospital, Capital Medical University, Beijing 100050, China; ^5^Key Laboratory for Neurodegenerative Disorders of the Ministry of Education, Beijing 100069, China; ^6^Center of Parkinson's Disease, Beijing Institute for Brain Disorders, Beijing 100053, China; ^7^China National Clinical Research Center for Neurological Diseases, Beijing 100050, China; ^8^Beijing Key Laboratory on Parkinson's Disease, Beijing 100053, China

## Abstract

*Objectives*. To explore effectiveness and mechanisms of electroacupuncture (EA) add-on treatment in Parkinson's disease (PD) patients.* Methods*. Fifty PD patients were randomly assigned to drug plus EA (D + EA) group and drug alone (D) group. Subjects in D + EA group received stimulation in points of bilateral fengfu, fengchi, hegu, and central dazhui. Participants were evaluated by scales for motor and nonmotor symptoms. Levels of neuroinflammatory factors and neurotransmitters in serum were detected.* Results*. EA add-on treatment remarkably reduced scores of Unified Parkinson's Disease Rating Scale (UPDRS) III and its subitems of tremor, rigidity, and bradykinesia and conspicuously decreased UPDRS III scores in patients with bradykinesia-rigidity and mixed types and mild severity. Depression and sleep disturbances were eased, which were reflected by decreased scores of Hamilton Depression Rating Scale, Pittsburgh Sleep Quality Index, and elevated noradrenaline level. Effects of EA add-on treatment on motor symptoms and sleep disturbances were superior to drug alone treatment, markedly improving life quality of PD patients. EA add-on treatment decreased nitric oxide level in serum.* Conclusions*. EA add-on treatment is effective on most motor symptoms and some nonmotor symptoms and is particularly efficacious in PD patients at early stage. Antineuroinflammation may be a mechanism of EA add-on treatment.

## 1. Background

Parkinson's disease (PD) is a common neurodegenerative disorder, whose prevalence is increasing with the aging of population. It is the second most common neurodegenerative disease after Alzheimer's disease [1]. PD was previously thought to be characterized by motor symptoms, including resting tremor, bradykinesia, rigidity, and gait and postural abnormalities. However, PD pathological stage based on the location of *α*-synuclein-immunopositive Lewy bodies [2] indicates the presence of numerous nonmotor symptoms [3, 4], including neuropsychiatric disturbance [5], abnormal sensation [6], sleep disorders [7], and autonomic dysfunction [8]. Drug therapy can alleviate the symptoms but fails to slow down and halt disease progression, and dopaminergic medication inevitably induces motor complications after long-term application [9, 10]. Although surgery partially relieves the symptoms, the efficacy over a long period of time has not yet been demonstrated, and it is expensive as well. Other therapeutic strategies, such as gene therapy and implantation, have been estimated for neuroprotection in the laboratory [11, 12], but the outcome is far from satisfactory and has a long journey to go for clinical therapy.

Acupuncture, as a traditional Chinese medicine, is drawing much attention due to its role in neurodegenerative disease. Although the therapeutic effect of acupuncture in PD is under debate, increasing evidence shows that it can alleviate motor symptoms and nonmotor symptoms, such as sleep [13] and pain and mood disturbances [14], and improve the quality of life in PD patients [15]. A group of experiments from animal models of PD indicate that acupuncture is effective in reducing oxidative stress [16], decreasing neuroinflammation with a major feature of microglial activation [17], stimulating release of neurotrophic factors [18], and regulating homeostasis of the network between cortex and striatum [19]. However, rare studies have investigated the effect of electroacupuncture (EA) on both motor and nonmotor symptoms and explored the potential mechanisms of the action of EA treatment in PD patients.

In the current study, we evaluated the effect of EA add-on treatment on both motor and nonmotor symptoms in PD patients by using related rating scales and explored the potential mechanisms of the therapeutic effect of EA by detecting the levels of neuroinflammatory factors, including nitric oxide (NO), tumor necrosis factor- (TNF-) *α*, interleukin- (IL-) 1*β*, and prostaglandin (PG) E_2_, and neurotransmitters, including dopamine (DA), acetylcholine (Ach), norepinephrine (NE), and 5-hydroxytryptamine (5-HT), in serum.

## 2. Methods

### 2.1. Ethics Statement

The protocol was approved by the Institutional Review Board of Beijing Tiantan Hospital. This study was performed in accordance with the ethical standards of the Helsinki Declaration and with the appropriate national regulations. Written informed consents were obtained from all participants.

### 2.2. Participants

Fifty PD patients diagnosed by neurologists were consecutively recruited from the Department of Geriatrics and Neurology, Beijing Tiantan Hospital, Capital Medical University, from May 2011 to May 2012. They were on a stable dose of anti-Parkinsonian medication for at least 2 months and did not report adverse events. The enrolled patient number was based on previous studies [20, 21]. Patients were diagnosed with PD according to UK Parkinson's Disease Society Brain Bank criteria [22]. All patients were advised not to change any of their anti-Parkinsonian medications during the study period. Patients whose prescriptions were changed during the study period or those who skipped more than 2 of the total 20 treatment sessions were eliminated from the study. Demographic information, including sex, age, Hoehn-Yahr (H-Y) Stage, disease duration, and daily dose of anti-Parkinsonian drugs, was recorded for all participants.

This pilot study was a randomized controlled clinical trial, in which PD patients were divided into two groups by completely randomized design. One group received additional EA treatment with the current drug treatment unchanged (drug plus EA (D + EA) group), and another group maintained the ongoing prescribed anti-Parkinsonian drugs with stable doses (drug alone (D) group). There were 30 patients in the D + EA group and 20 in the D group. Two patients in the D + EA group dropped out because of the change of anti-Parkinsonian drugs during the study period and failure to finish EA treatment. Finally, data from 28 patients in the D + EA group and 20 patients in the D group were analyzed.

Patients with secondary Parkinson syndromes, Parkinson plus syndromes, infectious disease in central and peripheral systems, dysarthria, severe psychiatric diseases affecting expression, malignant tumor, disability, and other serious somatic diseases were excluded.

### 2.3. Calculation of Levodopa Equivalent Dosages (LED)

LED was calculated based on the previously published methods: (regular levodopa dose × 1) + (levodopa controlled-release dose × 0.75) + [(regular levodopa dose + levodopa controlled-release dose × 0.75) × 0.25 + (pramipexole dose × 100) + (selegiline dose × 10)] [10].

### 2.4. EA Add-On Treatment Protocol

EA add-on treatment was performed by an experienced doctor in the Department of Traditional Chinese Medicine. Patients in the EA add-on treatment group received stimulation at 6 acupuncture points, including bilateral GB20 (Fengchi) and LI4 (Hegu) and central Du14 (Dazhui) and Du16 (Fengfu), which were selected based on previous studies [20, 21].

Sterile, stainless steel acupuncture needles with a diameter of 0.25 mm and length of 40 mm (Huatuo, China) were inserted obliquely into the desired points at a depth of 2.0–2.5 cm. After achieving Deqi, participants were then given electrical pulses of 9 V, 1 A, 9 W, and 100 Hz (KWD-808-II; Yingdi, China) for 30 minutes. Each course of EA treatment comprised 10 sessions and two courses were completed. EA treatment was performed once every 3 days and lasted 2 months.

### 2.5. Effect Assessments

Participants with PD were assessed by series of specialized scales before EA add-on treatment, including motor symptoms, motor complications, nonmotor symptoms, activity of daily living (ADL), and quality of life. Participants in the D + EA group were reassessed on the first day after completion of EA add-on treatment, and those in D group were reevaluated on the first day after 2 months of drug treatment. All participants were evaluated 12 hours after anti-Parkinsonian drugs had been withheld. Meanwhile, all the treatment information remained unknown to the professional assessor.

#### 2.5.1. Motor Symptoms and Motor Complications

Motor symptoms of PD patients were evaluated by Unified Parkinson's Disease Rating Scale (UPDRS) III, in which tremor evaluation was by Items 20 and 21, rigidity was by Item 22, and bradykinesia was by Items 23–26. According to the method for clinical phenotypes classification by Schrag et al. [23], participants were divided into tremor type, bradykinesia-rigidity type, and mixed type of PD. Severity of PD was assessed by H-Y Stage, based on which patients were divided into mild degree (Stages 1.0–2.0), moderate degree (Stages 2.5–3.0), and severe degree (Stages 4.0–5.0). Motor complications were assessed by UPDRS IV.

#### 2.5.2. Nonmotor Symptoms

Nonmotor symptoms of PD patients were screened by Nonmotor Symptoms Quest (NMSQ) followed by series of scales, including Montreal Cognitive Assessment (MoCA) and Mini-Mental State Examination (MMSE) for cognitive function, Hamilton Depression Scale- (HAMD-) 24 items for depression, Hamilton Anxiety Scale- (HAMA-) 14 items for anxiety, and Pittsburgh Sleep Quality Index (PSQI) for sleep quality.

#### 2.5.3. ADL and Quality of Life

ADL was evaluated by UPDRS II and ADL Scale. Quality of life was assessed by Parkinson's Disease Quality of Life Questionnaire- (PDQ-) 39 items.

### 2.6. Blood Collection

Anti-Parkinsonian drugs were withheld for 12–14 hours if the patient's condition allowed. Three milliliters of blood was taken under fasting conditions from the D + EA group before EA add-on treatment, and on the first day after completion of treatment, and from D group on the first day after 2 months of drug treatment. Blood samples were then centrifuged at 4°C at 3000 r/min for 10 minutes. Supernatants were preserved at −80°C until being used for detection.

### 2.7. Assays of Neuroinflammatory Factors in Serum

NO levels in serum from PD patients were detected by chemical colorimetric method. The A012 kit for NO assay was from Nanjing Jiancheng Biological Engineering Research Institute (China). The levels of TNF-*α*, IL-1*β*, and PGE_2_ in serum from PD patients were detected by enzyme-linked immunosorbent assay. The 1R350 kit for TNF-*α* and 1R040 kit for IL-1*β* were from Rapidbio (USA). The CSB-E07965h kit for PGE_2_ was from Cusabio (China).

### 2.8. Assays of Neurotransmitters in Serum

The levels of neurotransmitters, including DA, Ach, NE, and 5-HT in serum from PD patients, were measured by high-performance liquid chromatography. Phenomenex 150 × 2 mm, 150 × 3 mm chromatographic column, and liquid chromatography tandem mass spectrometry 6410 instrument were from Agilent (USA), and standard samples were from Sigma (St. Louis, MO, USA).

### 2.9. Data Analysis

Statistical analysis was performed with SPSS Statistics version 20.0 (Chicago, IL, USA). Scores for the above-mentioned scales and the levels of neuroinflammatory factors and neurotransmitters in serum were compared before and after EA add-on treatment in the D + EA group. The changes in the scores of the above-mentioned scales and the levels of neuroinflammatory factors and neurotransmitters in serum before and after EA add-on treatment also were compared between the D + EA and D groups. Continuous variables were presented as means ± standard deviations and compared by Student's *t*-test if they were normally distributed. Data were expressed as medians of the lower and upper quartiles and compared by Wilcoxon signed-rank test and Kruskal-Wallis test if they were not normally distributed. Discrete variables were compared by *χ* ^2^ test. *P* < 0.05 was considered statistically significant.

## 3. Results

### 3.1. Demographic Features

Demographics information, disease features, and LED were compared between the D + EA and D groups. There are no differences between the groups for demographic features and baseline information (Table 1).

### 3.2. Effects of EA Add-On Treatment

The effects of EA add-on treatment on PD patients were evaluated 12 hours after anti-Parkinsonian drugs had been withheld before and after two courses of EA add-on treatment were completed.

#### 3.2.1. Motor Function

*(1) Comparison before and after EA Add-On Treatment*. The effect of EA add-on treatment on motor symptoms was evaluated by UPDRS III. The scores of UPDRS III and its subitems, including tremor, rigidity, and bradykinesia, are all significantly decreased after EA add-on treatment (Table 2), indicating that EA add-on treatment has a significant effect on motor symptoms in PD patients.

After EA add-on treatment, the scores of UPDRS III in patients with bradykinesia-rigidity type and mixed type of PD are significantly decreased, but that in those with tremor type of PD is not significantly altered (Table 2).

H-Y Stage is not changed after EA add-on treatment (Table 2). Further analysis indicates that UPDRS III score is dramatically decreased in patients with mild PD and decreased in those with moderate PD; however, these differences are not significant. We cannot draw a conclusion in terms of the effect of EA add-on treatment on patients with severe PD from only one case collected (Table 2). 

*(2) Comparison between D + EA and D Groups*. Compared with the D group, motor symptoms of PD patients in the D + EA group are improved more clearly (Table 3). However, H-Y Stage is not markedly changed (Table 3).

#### 3.2.2. Motor Complications

The score of UPDRS IV reflecting motor complications in PD patients indicates no notable change before and after EA add-on treatment (0 points at both times) (*P* = 0.317).

#### 3.2.3. Nonmotor Symptoms

*(1) Comparison before and after EA Add-On Treatment*. As shown in Table 2, the scores of PSQI and HAMD in the D + EA group are significantly decreased after two courses of EA add-on treatment, implying that EA treatment significantly improves sleep quality and reduces depression. The increases in the scores of MoCA and MMSE verge on statistical significance, suggesting EA add-on treatment has the potential to improve cognitive function. EA add-on treatment fails to lower HAMA score, indicating no effect on ameliorating anxiety.

*(2) Comparison between D + EA and D Groups*. Compared with the D group, the PSQI score in the D + EA group is significantly reduced. There are no significant changes in the scores of HAMA, HAMD, MoCA, and MMSE between the D + EA and D groups (Table 3).

#### 3.2.4. ADL and Quality of Life

EA add-on treatment significantly reduces UPDRS II score from 10.6 ± 4.7 to 9.3 ± 3.8 points (*P* < 0.05), reflecting an improvement in ADL for PD patients. Meanwhile, EA add-on treatment conspicuously elevates PDQ-39 score from 149.2 ± 21.0 to 155.8 ± 17.8 points (*P* < 0.05), revealing an enhancement in quality of life for PD patients.

### 3.3. The Levels of Neuroinflammatory Factors in Serum

The levels of neuroinflammatory factors in serum were compared before and after EA add-on treatment. NO levels in serums from PD patients are significantly elevated after EA add-on treatment (Table 4). However, the amplitude of the increase in NO level in the D + EA group is notably lower than that in the D group (Table 5).

### 3.4. The Levels of Neurotransmitters in Serum

The levels of neurotransmitters in serum were compared before and after EA add-on treatment. There are obvious differences in the levels of Ach and NE, but not DA and 5-HT before and after EA add-on treatment (Table 4). The changes in neurotransmitter levels are not significant when comparing the D + EA and D groups (Table 5).

## 4. Discussion

Several studies on acupuncture treatment in PD patients have reported alleviation of tremor and walking difficulty; however, no data show a significant decrease in UPDRS III score [13, 24]. In the current study, the effect of EA add-on treatment on motor symptoms was evaluated by UPDRS III. Our results indicate that EA add-on treatment markedly reduces the scores of UPDRS III and its subitems, including tremor, rigidity, and bradykinesia (Table 2), indicating that EA add-on treatment is effective for most of the motor symptoms in PD patients. Further analysis reveals that the motor function reflected by UPDRS III score is conspicuously improved in patients with bradykinesia-rigidity type and mixed type of PD, but not in those with tremor type of PD after EA add-on treatment (Table 2). Conclusion in terms of the effect of EA add-on treatment on tremor type of PD cannot be drawn now because of only 3 patients with tremor type of PD recruited in the D + EA group in this investigation. We will enhance the sample size in the future with aim to draw a conclusion in this regard.

The effect of EA add-on treatment on the severity of PD was assessed by H-Y Stage. H-Y Stage is determined by the clinical manifestations which resulted from relatively long-term progression of PD; thus, 2 months of EA treatment is unlikely to change H-Y Stage notably (Table 2). However, further analysis demonstrates that motor function in patients with mild PD is markedly increased, which is indicated by the dramatically decreased UPDRS III score. Similarly, UPDRS III score is decreased in patients with moderate PD; however, the difference was not significant. These data reveal that the earlier the EA add-on treatment is adopted, the more effective it is. EA add-on treatment was administered to PD patients in the Traditional Chinese Medicine Clinic for 2 months; thus the majority of participants with mild and moderate PD had good treatment compliance. Only one patient with severe PD was recruited, which may make it difficult to make a conclusion about the effect of EA add-on treatment on patients with severe PD.

Motor symptoms of PD are caused by DA depletion in the striatum; therefore, restoration of striatal DA is suggested as a potential mechanism underlying the effectiveness of EA treatment on motor symptoms. Our data show that DA level is elevated, although not significantly after EA add-on treatment (Table 4). Several previous studies find no or poor correlation between DA level in the striatum and reduction of motor symptoms in PD patients after acupuncture [25, 26], implying that other mechanisms may be involved. Motor symptoms of PD are also associated with abnormal activity of *γ*-aminobutyric acid (GABA) neurons in the substantia nigra [27]. Jia et al. have reported that EA treatment decreases motor symptoms in an animal model by normalizing GABA activity in the substantia nigra, without increasing DA level in the striatum [26]. Moreover, positron emission tomography has shown that 5 weeks of combined treatment with madopar and acupuncture eases motor symptoms in PD patients by enhancing regional glucose metabolism in the parietal, temporal, and occipital lobes, thalamus, and cerebellum of the slightly affected hemisphere and the parietal and occipital lobes of the mildly affected hemisphere [28]. Functional magnetic resonance imaging suggests that acupuncture is effective by promoting neural responses in the regions beyond the substantia nigra, such as the caudate, thalamus, and putamen [29], which are also severely impaired in PD patients.

In the current study, EA add-on treatment is effective on motor symptoms and some nonmotor symptoms in PD patients. We have observed that depression is alleviated as HAMD score is significantly decreased after EA add-on treatment (Table 2), which is similar to the results of Cho et al. [20]. However, it remains unknown in the relationship between the level of neurotransmitters and depression in PD patients after EA add-on treatment. Raphe nucleus in midbrain and locus coeruleus in the pons are enriched in noradrenergic neurons, which extensively innervate the frontal and limbic lobes. According to the pathological stage of PD by Braak et al. [2], Lewy body formation and neuronal loss in the above areas result in a dramatic decrease in NE level, which may serve as an important neurochemical mechanism of PD depression. In a prospective 4-month follow-up study in 17 PD patients with depression, prescription with reboxetine, a selective NE reuptake inhibitor, eases depression and improves quality of life due to the reduction of NE reuptake, implying an elevated NE level after drug treatment [30]. Our result displays direct evidence showing an elevation of NE level in serum after 2 courses of EA add-on treatment (Table 4). The serotoninergic system, in which neurons are located in the midbrain with fibers innervating the reticular structure, limbic system, and prefrontal cortex, is impaired in PD patients. Degeneration in the above areas causes a decline in 5-HT level, which is responsible for mood disturbances. Drugs blocking the reuptake of 5-HT play a key role in the treatment of depression in PD patients. However, our data do not show an increase in 5-HT level in serum after EA add-on treatment (Table 4), which may be associated with rapid degradation of 5-HT in serum or insufficient time of EA add-on treatment.

Sleep disorders are one of the most common nonmotor symptoms of PD, compromising quality of life for patients. Our result implies that EA add-on treatment improves sleep quality as PSQI score is significantly decreased (Table 2). Sleep disturbances in PD involve the mesocorticolimbic dopaminergic system, a part of the thalamocortical arousal system, which originates from the ventral tegmental area and innervates the thalamus and hippocampus [31, 32]. DA plays a complex, yet to date not well known, role in the sleep-wake cycle in vertebrates, with an overall wakefulness-promoting effect [33]. Recent studies have highlighted the importance of DA in the circadian rhythm in mammals [34]. However, the current study shows that DA level in serum is not drastically elevated after EA treatment (Table 4), implying that there are other mechanisms underlying sleep disorders in PD. EA treatment may improve sleep quality by reducing motor symptoms and other nonmotor symptoms of PD [35, 36].

Cognitive decline is a result of long-term progression of PD pathology. The cognitive functions of participants in this study are impaired, which is indicated by the decreased MoCA score. EA add-on treatment increases both MoCA and MMSE scores (Table 2), which are close to statistical significance. The improvement in cognitive function is further supported by the enhancement of Ach level after EA add-on treatment (Table 4). Long-term EA add-on treatment may be needed in the future in order to see a dramatic effect on cognitive impairment.

In the current study, EA add-on treatment enhances ADL and quality of life as the scores for UPDRS III and PDQ-39 are elevated, respectively, which may have resulted from the relief of motor symptomsand nonmotor symptoms, including depression, sleep disturbances, and cognitive decline.

Compared with D group, motor function and sleep quality are remarkably improved in the D + EA group (Table 3), suggesting that EA add-on treatment is superior to drug alone in the above aspects. What are the underlying mechanisms? Neuroinflammation, characterized by microglial activation, serves as an engine driving PD progression [37]. In the substantia nigra, many endogenous and exogenous factors activate microglia and produce neuroinflammatory factors [38], such as NO, TNF-*α*, IL-1*β*, and PGE_2_, which cause dopaminergic neuronal death. The dead neurons release the substances, such as iron, aggregated *α*-synuclein, and neuromelanin, into the extracellular spaces and provoke chronic neuroinflammation by activating surrounding microglia [39], propagating progressive degeneration of dopaminergic neurons, and deterioration of motor symptoms of PD. Recently, the significance of neuroinflammation in PD pathology has extended beyond the substantia nigra, causing damage to other regions relevant to nonmotor symptoms [40, 41]. Among the neuroinflammatory factors released by activated microglia [38], NO is one of the pivotal factors in neuroinflammatory event. Meanwhile, increasing evidence indicates that the NO level in serum in PD patients is associated not only with PD but also with the severity of the disease [42, 43]. There is a report suggesting that peripheral inflammation increases the deleterious effect of neuroinflammation on the nigrostriatal dopaminergic system in brain through the multiple mechanisms [44], for example, probably by different mechanisms: peripheral inflammation contributes to the damage and delays the restoration of the compromised BBB and maintains the activation of microglia; circulating monocytes enter the brain parenchyma to further enhance the inflammatory response under conditions of disrupted BBB [45]. In the current study, although NO level in serum is increased after EA add-on treatment (Table 4), the amplitude of the increase is significantly lower than that in the D group (Table 5), indicating that EA add-on treatment may slow down the acceleration of neuroinflammation by reducing NO release. NO readily reacts with superoxide, also produced by activated microglia, to produce highly toxic peroxynitrite anions (ONOO^−^). ONOO^−^ causes DNA base modifications and strand breaks [46] and induces disruption of enzyme function and protein structure integrity by covalently modifying tyrosine residues, provoking cellular apoptosis or necrosis [47]. Importantly, not all neuroinflammatory factors are significantly enhanced in disease conditions; however, some of them work together and amplify the neuroinflammatory effect of each other. It is found that TNF-*α* and IL-1 potentiate an ongoing neuroinflammatory response by releasing NO [48, 49]. In the current study, although the levels of IL-1*β*, TNF-*α*, and PGE_2_ are not substantially decreased (Table 4), EA add-on treatment may slow down the progression of neuroinflammation because of its inhibitory effect on each neuroinflammatory factor. This may cause either additive or synergistic antineuroinflammatory effects, by which EA add-on treatment improves motor symptoms and some nonmotor symptoms. Thus, antineuroinflammation may be a potential mechanism underlying the effectiveness of EA add-on treatment on both motor symptoms and nonmotor symptoms in PD patients.

This pilot study has several limitations, most notably its relatively insufficient sample size and the failure to exclude the specific placebo effects. Although no definite conclusion can be drawn from our results, the patients in our trial who were given EA add-on treatments show significant improvement in a variety of symptoms, indicating a potential benefit of this treatment as an adjunct in PD patients.

## 5. Conclusion

On the one hand, EA add-on treatment dramatically reduces motor symptoms of PD patients, as indicated by the decrease in UPDRS III score, especially in those with rigidity-bradykinesia type and mixed type of PD, and those with mild PD. However, definitive conclusions can only be drawn after a larger study with long-term follow-up. On the other hand, EA add-on treatment eases nonmotor symptoms, especially sleep quality and depression which was reflected by the increased level of NE. However, long period of EA add-on treatment is still needed in the future in order to see its dramatic effect on cognitive impairment. The effect of EA add-on treatment on motor symptoms and sleep quality exceeds that of drug treatment alone. Accordingly, EA add-on treatment markedly improves ADL and quality of life of PD patients. Antineuroinflammation by decreasing the elevation of NO level may be a potential mechanism underlying the effectiveness of EA add-on treatment on both motor symptoms and nonmotor symptoms in PD patients.

## Figures and Tables

**Table 1:** Demographic features and baseline characteristics of patient in the D + EA and D groups.

	D + EA group	D group	*P*
Gender, male/total (*n*)	13/28	9/20	0.922^c^
Age (years, mean ± SD)	62.1 ± 8.7	59.1 ± 12.4	0.334^a^
Duration (years, mean ± SD)	2.9 ± 2.9	2.7 ± 2.3	0.719^a^
Tremor type (*n*)	3	4	0.628^c^
Rigidity-bradykinesia type (*n*)	8	5	0.784^c^
Mixed type (*n*)	17	11	0.692^c^
H-Y Stage (stage, mean ± SD)	2.0 ± 0.7	2.0 ± 0.8	0.907^a^
LED (mg, mean ± SD)	104.1 ± 253.2	160.6 ± 260.0	0.376^a^
UPSRS III (points, mean ± SD)	25.6 ± 2.8	23.3 ± 13.0	0.583^a^
PSQI [points, median (quartile)]	21.0 (18.0~25.3)	16.5 (13.8~23.0)	0.088^b^
HAMD [points, median (quartile)]	12.0 (7.0~19.0)	7.5 (5.0~17.8)	0.098^b^
HAMA [points, median (quartile)]	13.5 (10.0~16.0)	9.0 (6.3~14.0)	0.139^b^
MMSE [points, median (quartile)]	29.0 (28.0~30.0)	28.0 (25.0~29.0)	0.065^b^
MoCA [points, median (quartile)]	25.0 (22.0~28.0)	23.5 (17.3~27.0)	0.252^b^

^a^Values are means ± standard for Student's *t*-test.

^b^Values are median (lower quartile, upper quartile) for the Kruskal-Wallis test.

^c^Values are discrete variables for the *χ* ^2^ test.

LED: levodopa equivalent dose.

**Table 2:** Scores of motor symptoms and nonmotor symptoms before and after EA add-on treatment in the D + EA group, and before and after 2 months in the D group.

	D + EA group		*P*	D group		*P*
	Before EA	After EA		Before 2 months	After 2 months	
*Motor symptoms*						
UPDRS III [points (mean ± SD)]	25.6 ± 2.8	20.6 ± 2.7	0.000^*∗∗*a^	23.3 ± 13.0	22.5 ± 12.1	0.061^a^
Subitems of UPDRS III						
Tremor [points (mean ± SD)]	4.9 ± 4.4	3.4 ± 3.9	0.001^*∗∗*a^	4.5 ± 3.1	4.3 ± 2.8	0.353^a^
Rigidity [points (mean ± SD)]	4.6 ± 3.4	3.6 ± 3.2	0.004^*∗∗*a^	4.2 ± 4.1	4.0 ± 3.9	0.070^a^
Bradykinesia [points (mean ± SD)]	9.0 ± 6.3	7.4 ± 5.9	0.000^*∗∗*a^	7.8 ± 5.3	7.4 ± 5.2	0.072^a^
Gait and postural abnormality [points (mean ± SD)]	2.8 ± 1.3	2.7 ± 1.6	0.788^a^	3.6 ± 2.5	3.4 ± 2.2	0.168^a^
UPDRS III of different PD clinical phenotypes						
Tremor type [points (mean ± SD)]	14.0 ± 6.5	10.0 ± 3.0	0.373^a^	9.8 ± 6.8	9.7 ± 6.7	0.363^a^
Rigidity-bradykinesia type [points (mean ± SD)]	17.8 ± 5.0	13.6 ± 3.9	0.027^*∗*a^	21.8 ± 5.7	19.8 ± 2.2	0.391^a^
Mixed type [points (mean ± SD)]	31.3 ± 3.1	25.8 ± 3.5	0.000^*∗∗*a^	32.0 ± 10.6	30.5 ± 9.8	0.062^a^
UPDRS III scores of different PD severity						
Mild degree [points (mean ± SD)]	20.2 ± 2.6	14.7 ± 2.0	0.000^*∗∗*a^	16.6 ± 9.1	15.8 ± 8.3	0.056^a^
Moderate degree [points (mean ± SD)]	33.5 ± 3.9	29.1 ± 4.1	0.055^a^	33.4 ± 11.5	31.1 ± 10.1	0.090^a^
H-Y Stage (stage, mean ± SD)	2.0 ± 0.7	1.9 ± 0.7	0.096^a^	2.1 ± 0.8	2.0 ± 0.8	1.000^a^
Nonmotor symptoms						
PSQI [points, median (quartile)]	21.0 (18.0~25.3)	20.0 (18.0~22.0)	0.018^*∗*b^	16.5 (13.8~23.0)	16.5 (13.3~22.0)	0.850^b^
HAMD [points, median (quartile)]	12.0 (7.0~19.0)	12.0 (7.0~18.0)	0.034^*∗*b^	7.5 (5.0~17.8)	7.5 (5.0~14.5)	0.870^b^
HAMA [points, median (quartile)]	13.5 (10.0~16.0)	12.5 (7.3~14.0)	0.235^b^	9.0 (6.3~14.0)	8.5 (6.0~13.8)	0.694^b^
MMSE [points, median (quartile)]	29.0 (28.0~30.0)	29.0 (28.0~30.0)	0.063^b^	28.0 (25.0~29.0)	28.0 (25.0~29.0)	0.893^b^
MoCA [points, median (quartile)]	25.0 (22.0~28.0)	25.0 (22.0~28.0)	0.083^b^	23.5 (17.3~27.0)	23.5 (17.3~27.0)	0.876^b^

^a^Values are means ± standard for the Paired *t*-test.

^b^Values are median (lower quartile, upper quartile) for the Wilcoxon signed-rank test.

^*∗*^ *P* < 0.05; ^*∗∗*^ *P* < 0.01.

**Table 3:** Changes in the scores of motor and nonmotor symptoms in the D + EA and D groups.

	D + EA group (before and after EA)	D group (before and after 2 months)	*P*
UPDRS III [points (mean ± SD)]	4.9 ± 4.8	2.3 ± 3.0	0.036^*∗*a^
H-Y Stage [stage, median (quartile)]	0.0 (0.0~0.0)	0.0 (0.0~0.0)	0.135^b^
PSQI [points, median (quartile)]	1.0 (0.0~2.0)	0.0 (0.0~0.0)	0.034^*∗*b^
HAMD [points, median (quartile)]	0.0 (0.0~1.0)	0.0 (0.0~0.0)	0.360^b^
HAMA [points, median (quartile)]	0.0 (0.0~0.0)	0.0 (0.0~0.0)	0.989^b^
MMSE [points, median (quartile)]	0.0 (0.0~0.0)	0.0 (0.0~0.0)	0.081^b^
MoCA [points, median (quartile)]	0.0 (−1.0~0.0)	0.0 (0.0~0.0)	0.079^b^

^a^Values are means ± standard for Student's *t*-test.

^b^Values are median (lower quartile, upper quartile) for the Kruskal-Wallis test.

^*∗*^ *P* < 0.05.

**Table 4:** Levels of neuroinflammatory factors and neurotransmitters in serum before and after EA add-on treatment in the D + EA group, and before and after 2 months in the D group.

	D + EA group		*P*	D group		*P*
	Before EA	After EA		Before 2 months	After 2 months	
Neuroinflammatory factors						
NO [mmol/L, median (quartile)]	29.927 (17.153–51.460)	83.108 (63.869–106.081)	0.002^*∗∗*^	29.927 (16.970–50.182)	116.891 (93.598–145.946)	0.000^*∗∗*^
IL-1*β* [pg/mL, median (quartile)]	4.160 (2.445–6.187)	6.647 (4.191–9.817)	0.055	7.527 (5.536–8.839)	9.170 (5.025–12.279)	0.204
PGE_2_ [pg/mL, median (quartile)]	10.105 (4.167–50.510)	9.745 (6.876–16.922)	0.292	6.081 (4.612–13.875)	10.364 (7.449–12.968)	0.232
TNF-*α* [pg/mL, median (quartile)]	63.715 (42.480–70.383)	70.741 (44.571–122.699)	0.218	65.407 (61.229–68.284)	81.852 (47.271–114.851)	0.058
Neurotransmitters						
NE [*μ*g/mL, median (quartile)]	0.132 (0.088~1.021)	1.453 (1.216~1.801)	0.002^*∗∗*^	0.114 (0.096–1.109)	0.799 (0.423–1.382)	0.093
Ach [nmol/L, median (quartile)]	0.007 (0.004~0.016)	0.011 (0.009~0.014)	0.024^*∗*^	0.008 (0.005–0.012)	0.016 (0.009–0.021)	0.445
DA [*μ*g/mL, median (quartile)]	1.688 (0.002~2.737)	1.959 (0.764~3.037)	0.073	1.780 (1.165–2.593)	1.797 (1.162–2.691)	0.646
5-HT [*μ*g/mL, median (quartile)]	0.236 (0.143~1.313)	0.486 (0.129~1.205)	0.761	0.232 (0.199–0.301)	0.307 (0.169–0.608)	0.241

Values are median (lower quartile, upper quartile) for the Wilcoxon signed-rank test.

^*∗*^ *P* < 0.05; ^*∗∗*^ *P* < 0.01.

**Table 5:** Changes in the levels of neuroinflammatory factors and neurotransmitters in serum in the D + EA and D groups.

	D + EA group (after and before EA)	D group (after and before 2 months)	*P*
Neuroinflammatory factors			
NO [mmol/L, median (quartile)]	53.181 (6.421~64.505)	80.494 (62.154~107.569)	0.003^*∗∗*^
IL-1*β* [pg/mL, median (quartile)]	2.083 (−0.938~5.337)	2.525 (−3.358~4.034)	0.425
PGE_2_ [pg/mL, median (quartile)]	1.373 (−35.700~6.699)	1.436 (−2.378~6.738)	0.215
TNF-*α* [pg/mL, median (quartile)]	8.643 (−22.139~62.882)	20.537 (−17.089~52.414)	0.773
Neurotransmitters			
NE [*μ*g/mL, median (quartile)]	1.234 (0.734~1.512)	1.208 (0.609~1.878)	0.808
Ach [nmol/L, median (quartile)]	0.007 (0.002~0.011)	0.008 (0.003~0.015)	0.603
DA [*μ*g/mL, median (quartile)]	1.429 (0.518~3.035)	1.795 (1.161~2.683)	0.764
5-HT [*μ*g/mL, median (quartile)]	0.077 (−0.294~0.506)	0.703 (0.109~0.981)	0.055

Values are median (lower quartile, upper quartile) for the Kruskal-Wallis test.

^*∗∗*^ *P* < 0.01.

## Abbreviations

PD: Parkinson's disease
EA: Electroacupuncture
NO: Nitric oxide
TNF-*α*: Tumor necrosis factor-*α*
IL-1*β*: Interleukin-1*β*
PGE_2_: Prostaglandin E_2_
DA: Dopamine
Ach: Acetylcholine
NE: Noradrenaline
5-HT: 5-Hydroxytryptamine
H-Y: Hoehn-Yahr
LED: Levodopa equivalent dosages
NMSQ: Nonmotor Symptoms Quest
MoCA: Montreal Cognitive Assessment
MMSE: Mini-Mental State Examination
HAMD: Hamilton Depression Scale
HAMA: Hamilton Anxiety Scale
ADL: Activity of daily living
PDQ-39: Parkinson's Disease Quality of Life Questionnaire-39 items.
